# Lack of prognostic significance of p16 and p27 after radical prostatectomy in hormone-naïve prostate cancer

**DOI:** 10.1186/1477-5751-11-2

**Published:** 2012-01-05

**Authors:** Panagiotis J Vlachostergios, Foteini Karasavvidou, Grigorios Kakkas, Kassiani Kapatou, Ioannis Gioulbasanis, Danai D Daliani, George Moutzouris, Christos N Papandreou

**Affiliations:** 1 Department of Medical Oncology, University Hospital of Larissa, University of Thessaly School of Medicine, Larissa, Greece; 2 Department of Pathology, University Hospital of Larissa, University of Thessaly School of Medicine, Larissa, Greece; 3 Department of Urology, University Hospital of Larissa, University of Thessaly School of Medicine, Larissa, Greece

## Abstract

**Background:**

Loss of normal cell cycle control is an early event in the evolution of cancer. The expression of cyclin-dependent kinase (CDK) inhibitors p16 and p27 has been previously associated with progression of prostate cancer (PC). 70 patients diagnosed with early stage PCwere treated with radical prostatectomy (RP) at our institution and their tumor specimens were immunohistochemically evaluated for expression of p16 and p27. Available clinical data of time to PSA recurrence were correlated with the examined parameters and combined with pre-operative PSA level, Gleason score and pathological TNM (pT) stage assessment.

**Results:**

Nuclear overexpression of p16 was not associated with time to biochemical failure (BF) (p = 0.572). Same was the case for nuclear p27 overexpression (p *= *1.000). Also, no significant correlations were found between either p16 or p27, and pre-operative PSA level, pT stage and Gleason grade. pT stage emerged as the only independent prognostic factor for biochemical recurrence (p = 0.01).

**Conclusions:**

These data question previously reported data supporting the prognostic relevance of both p16 and p27 proteins in early PC.

## Background

There is increasing evidence that cell cycle regulators are disrupted in human cancers [1]. The cell cycle is governed by cyclin-dependent kinases (CDKs), the activities of which are regulated by binding of positive effectors, the cyclins [2]; by negative regulators, the CDK inhibitors [3] and by phosphorylation and dephosphorylation events.

p16 protein, encoded by the INK4A gene mapping to the 9p21 region [4,5] acts as a negative cell cycle regulator. Specific mechanisms may contribute to p16 altered expression, overcoming p16-mediated tumor suppressor activities. Unlike other primary tumors, INK4A inactivation, through deletions, mutations, or promoter methylation, seems to be an infrequent event in primary prostate cancer (PC) [6]. In contrast, the more frequent alterations of p16 in metastatic disease suggest that this might be a late event during the progression of some prostate carcinomas. It seems that p16 is overexpressed rather than lost in a large proportion of prostate carcinomas as p16 protein expression was increased in a majority of adenocarcinomas of the prostate and in prostate intra-epithelial neoplasia (PIN) when compared with surrounding benign glands [7]. Loss of transcriptional repression in the presence of inactivating mutations in the retinoblastoma (RB) gene is the most well-defined mechanism of p16INK4A overexpression [8]. p16 expression in premalignant lesions and carcinomas but not in normal or benign tissues implies a role of p16INK4A detection in the diagnosis of difficult cases of PIN and PC [9].

p27Kip1 is another CDK inhibitor that negatively regulates cell proliferation by mediating cell cycle arrest in G1. It has been suggested that decreased expression of the p27Kip1 protein may contribute to the development of human malignancies due to loss of critical anti-proliferative mechanisms. Unlike other CDK inhibitor genes, the p27Kip1 gene is rarely mutated in human cancers [10]. Instead, loss of p27Kip1 appears to occur through accelerated degradation by the ubiquitin-proteasome pathway. Loss of p27 expression in human PC cells was correlated with advancing histological aggressiveness, implicating deregulation of p27 in prostate tumor progression [11,12]. Down-regulation of expression of p27Kip1 in neoplastic progression from pre-invasive lesions through invasive carcinoma and metastases occurs in the early phases of neoplastic PC evolution [13].

There seems to be a close molecular association between these two CDK inhibitor proteins as p16INK4A-mediated growth inhibition may occur only when cyclin E/Cdk2 complexes are inactivated concurrently by p27Kip1 [14]. Reversely, loss of p16 seems to contribute to p27 sequestration by cyclin D1-CDK 4 complexes and confers poor prognosis in hepatocellular carcinoma [15]. Progressive and sustained increases in both p27 and p16 protein expression are considered to occur as mid-to-late events during evolution of PC [16].

In this study we sought to determine whether there is a clinically relevant interrelation based on immunohistochemical detection of p16 and p27 in radical prostatectomy (RP) specimens of hormone-naïve PC patients. Associations between p16 and p27 phenotypes and clinico-pathological variables were also studied to further define their potential use as prognostic indicators of biochemical failure (BF) in early PC.

## Methods

### Patients

The study enrolled patients over 18 years old with histologically newly diagnosed, early stage PC, admitted to the Department of Urology of our Institution. All patients of the study underwent an open retropubic RP. Patients were hormone- and treatment- naïve at the time of surgery. No history of previous reproductive or endocrine diseases was reported. Written informed consent was provided by all patients before study entry. The study was approved by the Ethics and Scientific Committees of our Institution. Patient demographics (age) as well as clinico-pathological parameters, including pre-operative PSA level, pathological TNM (pT) stage and Gleason score of the primary tumor, PSA recurrence and survival data were recorded.

The RP specimens were fixed in 10% buffered formalin solution and embedded in paraffin blocks. The complete sampling scheme with routine sections was used. H&E - stained tissue sections from 70 patients were examined by a single, blinded histopathologist and evaluation of histopathological characteristics was made according to recommendations of the 2004 World Health Organization (WHO) - sponsored International Consultation on Prediction of Patients Outcome in Prostate Cancer meeting [17]. Cases were grouped into 2 Gleason groups, low (≤ 7, 7 = 3+4; *n *= 50) and high (≥ 7, 7 = 4+3; *n *= 20) as there were no lower Gleason score (2, 3, 4) samples based on the established 3-group histopathological criteria of current literature (low, medium and high). Cases were also grouped according to pT stage into either organ confined disease (pT ≤ 2; *n *= 42) or advanced tumors extending beyond the prostatic capsule (pT > 2; *n *= 28). Patients were categorized into 3 sub-groups according to pre-operative PSA level (< 5 ng/ml, *n *= 14; 5-10 ng/ml, *n *= 48 and > 10 ng/ml, *n *= 8). Intermediate risk PC is generally considered with a PSA of 10-20 ng/ml, but we explored whether there is any difference in PSA relapse-free survival between subgroups of < 5, 5-10 and > 10 ng/ml within our cohort of low-to-intermediate risk for PSA recurrence, as indicated by the range of pre-operative PSA values (2.8-23.9 ng/ml). The majority of patients featured a negative lymph node status (*n *= 53). The latter was not included in the statistical analyses due to missing information regarding a significant number of patients (*n *= 11 or 15.7%). Patients' clinical and pathological characteristics are depicted in Table 1.

**Table 1:** Patients' clinical and pathological characteristics

Variable	Subgroup	*n *(%)
age (years)	≤ 65	31 (44.3)
	
range 47-75	> 65	39 (55.7)

pre-op PSA (ng/ml)	< 5	14 (20.0)
	
range 2.8-23.9	5-10	48 (68.6)
	
	> 10	8 (11.4)

pT stage	≤ 2	42 (60.0)
	
	> 2	28 (40.0)

Gleason score	≤ 7 (3+4)	50 (46.8)
	
	≥ 7 (4+3)	20 (44.2)

pre-op PSA, pre-operative PSA; pT stage, pathologic TNM stage

### Immunohistochemistry

Sections (4 μm) from selected paraffin blocks of each case were obtained. Sections were deparaffinised in xylene and rehydrated through decreasing alcohols. Antigen unmasking for p16 and p27 was achieved by boiling sections in Trilogy reagent (Cell Marque, Rocklin, Calif) for a total of 1 hour in a commercially available steamer. After quenching endogenous peroxidase with 3% hydrogen peroxide solution for 10 min, slides were incubated at room temperature for 30 minutes with the following primary mouse monoclonal antibodies: against p16 [clone E6H4, mouse monoclonal, CINTEC, ready to use (RTU)] and anti-p27 (clone 5X53G8, mouse monoclonal, DAKO, Denmark, in 1:50 dilution). Staining was developed with substrate chromogen solution (EnVision, DAKO, Glostrup, Denmark) and diaminobenzidine for 10 minutes. Slides were counterstained with Harris hematoxylin for 1 minute, dehydrated, and mounted with DPX solution. p16 immunostaining was nuclear. The extent of immunostaining was categorized into 4 groups according to the percentage of immunostained neoplastic cells: negative (0), less than 30% (1), 30% to 70% (2) and more than 70% (3). p27 immunostaining was nuclear. The extent of immunostaining was categorized into 2 groups according to the percentage of immunostained neoplastic cells: less than 70% (1), and more than 70% (2). Groups 1, 2 and 3 of p16 expression were merged together for statistical analysis. Immunohistochemical reaction was glandular for all tested parameters (p16, p27). The normal adjacent prostate gland was used as negative internal control marker for p16 expression and positive control for p27 expression.

### Study endpoints

Our objective was to investigate possible interrelations between immunohistochemical expression of p16 and p27 as well as their potential correlations with pre-operative PSA level, Gleason score and pT stage in patients with hormone naïve PC undergoing RP. We further examined the putative prognostic role of these parameters in association with time to PSA relapse. The response variable, time to BF, was defined as the time from RP to the time of the first detectable (non-zero) PSA measurement. To confirm PSA relapse, three consecutive increases of PSA were required; however, the time of relapse was defined as the time of the first detectable PSA measurement [18].

### Statistical analyses

The Fisher's, Pearson's Chi-squared and χ^2 ^tests were used to explore associations between p16, p27 expression patterns and pre-operative PSA level, Gleason score, and pT stage. The Kaplan-Meier method was used to determine the effect of each categorical variable on biochemical relapse-free survival, and the log-rank test was used to compare recurrence-free survival differences within each variable. For PSA recurrence-free survival analysis at the multivariate level the Cox proportional hazards model was used to estimate hazard ratios (HR) with 95% confidence intervals (CI). Statistical significance was determined by using two-tailed p-values and was reported at p < 0.05 level. Statistical analysis was performed using SPSS (SPSS for Windows, version 15.0, SPSS, Chicago, IL).

## Results

Thirty-seven (53%) patients developed PSA recurrence during follow up, thirty-three (47%) did not have a PSA relapse and two patients (2.8%) expired. The estimated median follow up time, as calculated by the reverse Kaplan-Meier method was 30 months (range 12-86) while the median time to BF was 56 months (range 1-74).

According to level of p16 expression, patients were divided into a group of negative nuclear staining (*n *= 54, 77.1%) and another of positive nuclear p16 immunohistochemical expression (*n *= 16, 22.9%). In univariate analysis, we observed no significant association of p16 with Gleason score (p = 0.565) or pT stage (p = 0.394) (Table 2). Further, there were no statistically significant correlations between p16 and pre-operative PSA levels (Table 2). The expression of p16 was not associated with time to BF (p = 0.572).

**Table 2:** Correlations between levels of p16, p27 expression and clinico-pathological characteristics

Variable		p16						p27					
		
		low		high		*n*	*p value*	low		high		*n*	*p value*
							
		*N*	%	*n*	%			*n*	%	*n*	%		
**pre-op PSA**	**< 5**	10	71.4	4	28.6	14	0.252	12	85.7	2	14.3	14	0.397
	
	**5-10**	36	75	12	25	48	0.104	39	81.2	9	18.8	48	0.215
	
	**> 10**	8	100	0	0	8	0.180	8	100	0	0	8	0.534

**Gleason** **score**	**≤ 7 (3+4)**	21	72.4	8	27.6	29	0.565	25	86.2	4	13.8	29	1.000
				
	**≥ 7 (4+3)**	33	80.5	8	19.5	41		34	82.9	7	17.1	41	

**pT stage**	**≤ 2**	34	80.9	8	19.1	42	0.394	37	88.1	5	11.9	42	0.328
				
	**> 2**	20	71.4	8	28.6	28		22	78.6	6	21.4	28	

**PSA relapse**	**no**	24	72.7	9	27.3	33	0.572	27	81.8	6	18.2	33	1.000
				
	**yes**	30	81.1	7	18.9	37		31	83.8	6	16.2	37	

pre-op PSA, pre-operative PSA; pT stage, pathologic TNM stage

p27 expression was distributed in two groups of either low (*n *= 59, 84.3%) or high (*n *= 11, 15.7%) nuclear immunoreactivity. p27 positivity did not correlate with neither Gleason score (p = 1.000) nor pT stage (p = 0.328) in univariate analysis (Table 2). p27 was not predictive of PSA biochemical recurrence (p = 1.000) and there were no statistically significant correlations between p27 and pre-operative PSA levels (Table 2). p16 and p27 expression did not present any statistically significant interrelation (p = 0.705).

**Figure 1:**
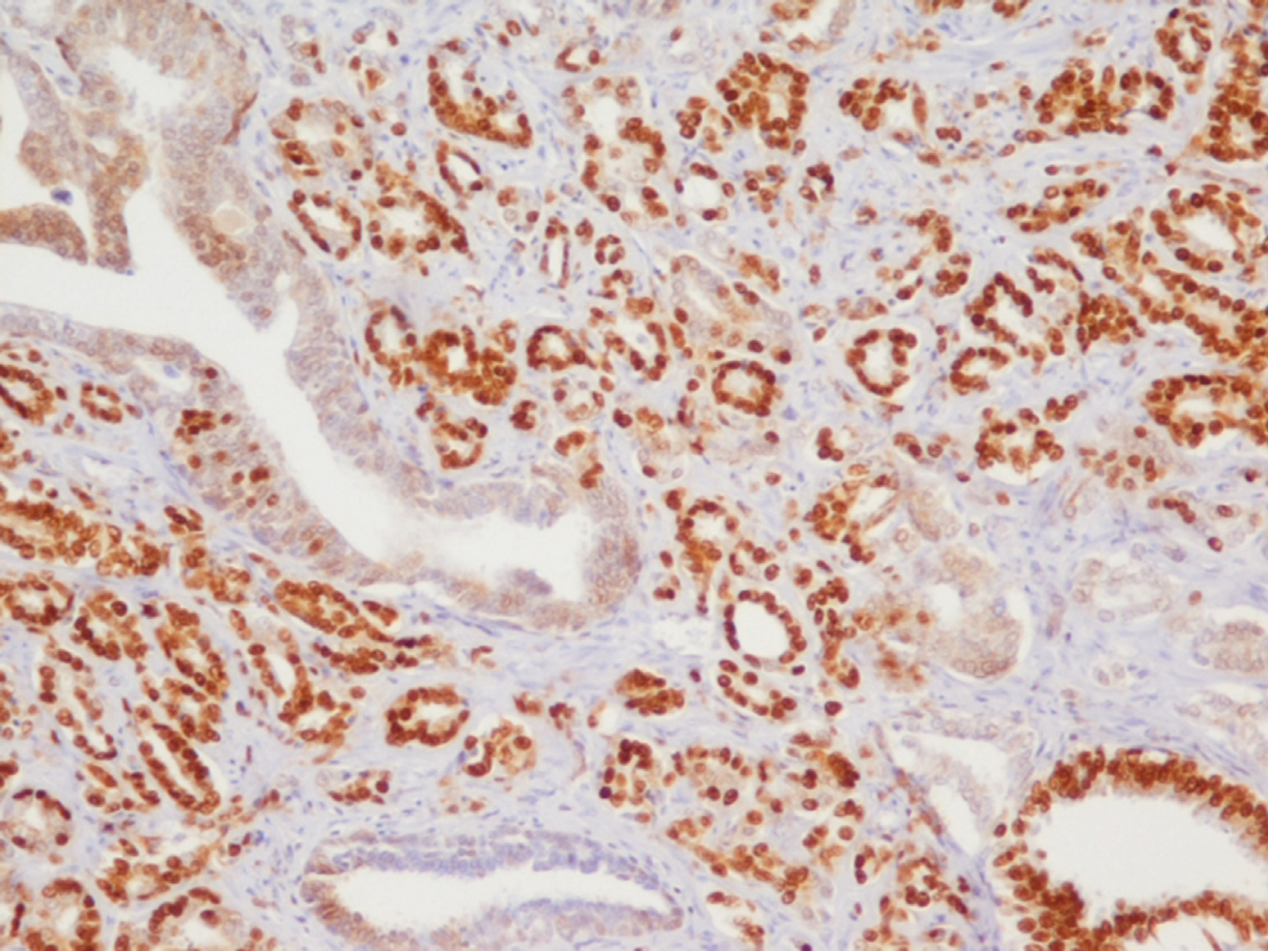
**Prostate adenocarcinoma Gleason pattern 3 with high p27 expression**. Strong nuclear immunohistochemical staining for p27, high expression (> 70%). p27 × 100.

**Figure 2:**
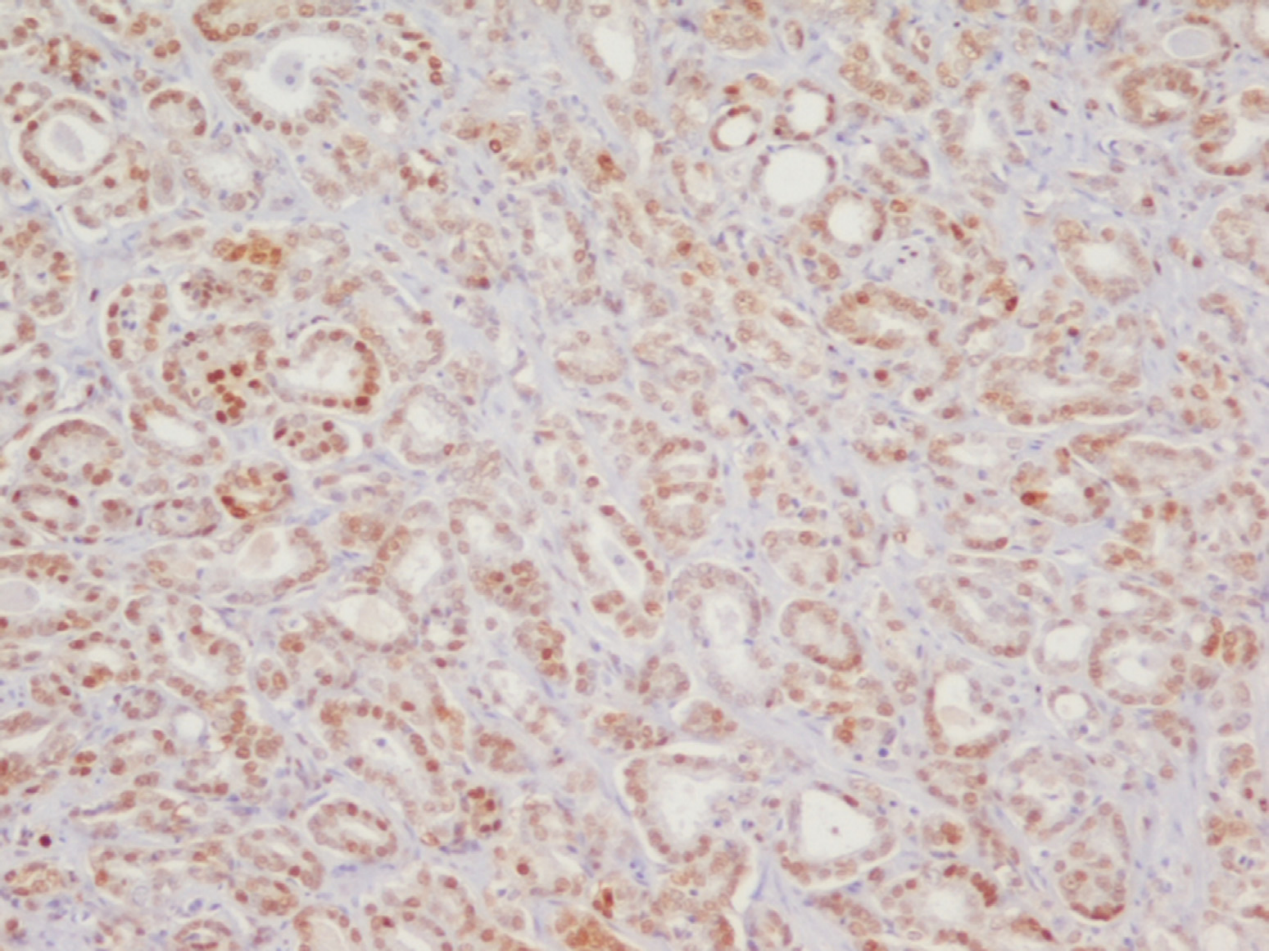
**Prostate adenocarcinoma Gleason pattern 3 with low p27 expression**. Nuclear immunohistochemical staining for p27, low expression (< 70%). p27 × 100.

**Figure 3:**
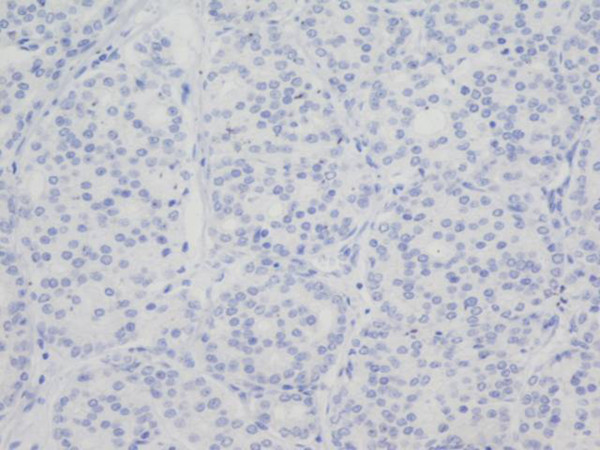
**Prostate adenocarcinoma Gleason pattern 4 with no p16 expression**. Negative immunoreactivity for p16. p16 × 200.

**Figure 4:**
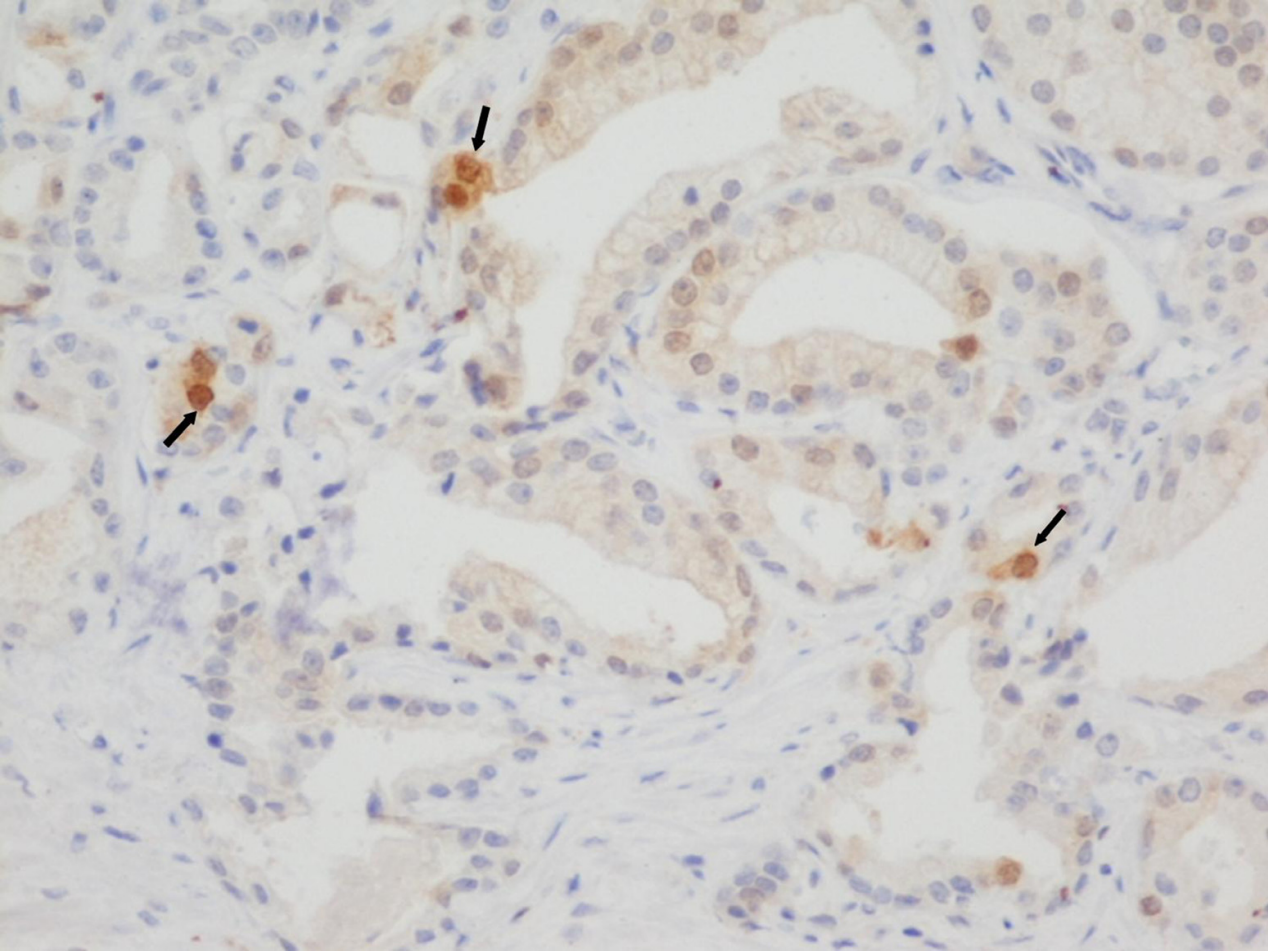
**Prostate adenocarcinoma Gleason pattern 3 with p16 expression**. Nuclear immunohistochemical staining for p16 (arrows). p16 × 200.

Immunohistochemical expression patterns of p16 and p27 are depicted in Figures 1, 2 and 3, 4 respectively.

pT stage and Gleason score were directly interrelated (p = 0.028) and were both inversely related with time to BF (p < 0.001 and p = 0.008 respectively). pT stage was directly associated with pre-operative PSA levels < 5 ng/ml (p = 0.016), 5-10 ng/ml (p = 0.007) and > 10 ng/ml (p = 0.054). There were no statistically significant correlations between pre-operative PSA levels and time to BF [PSA < 5 ng/ml (p = 0.311), 5-10 ng/ml (p = 0.309) and > 10 ng/ml (p = 0.167)] or grade [PSA < 5 ng/ml (p = 0.123), 5-10 ng/ml (p = 0.125) and > 10 ng/ml (p = 0.823)].

In multivariate analysis, only pT stage emerged as independent prognostic factor of biochemical relapse (p = 0.01) (Table 3).

**Table 3:** Multivariate Cox regression analysis

Variable	Hazard ratio	95% Confidence interval		*P *value
			
		Lower limit	Upper limit	
**pre-op PSA < 5 ng/ml**	0.683	0.155	2.681	0.766

**pre-op PSA 5-10 ng/ml**	0.838	0.262	3.013	0.639

**pre-op PSA > 10 ng/ml**	1.463	0.332	6.449	0.615

**Gleason score**	1.448	0.586	3.581	0.423

**pT stage**	3.354	1.342	8.386	0.010

**p16**	0.643	0.260	1.592	0.340

**p27**	1.065	0.403	2.813	0.899

pre-op PSA, pre-operative PSA; pT stage, pathologic TNM stage

## Discussion

In this study we have simultaneously examined the immunohistochemical expression of p16 and p27 in RP specimens of hormone naïve PC patients and reported the absence of a prognostic role for p16 and p27 in primary PC, as we observed no correlations with most important, firmly established clinico-pathological parameters, including pre-operative PSA, tumor stage and grade, even at the univariate level. In multivariate analysis including p16, p27, pre-operative PSA level, tumor stage and Gleason grade, only pT stage retained its importance in predicting PSA recurrence after RP. In fact, the risk of PSA relapse was approximately 3.3 fold greater in patients with advanced pT stage than in men with early pT stage disease.

Both histological markers have been previously studied in early PC, often with conflicting results regarding their putative prognostic relevance. An initial immunohistochemical study of p16 expression in 88 early PC patients reported a significant univariate association of p16 with a higher pre-treatment PSA level and a sooner time to PSA relapse after RP [6], although p16 was not associated with Gleason grade or stage. Unlike our study population, this cohort followed a higher threshold of detection (> 5%) for characterization of p16 nuclear staining in tumor cells. Also, a significant portion of the cohort (34 patients or 39%) was treated with neo-adjuvant androgen ablation, which seemed to enhance p16 expression. In multivariate analysis adjusted for tumor grade, pre-treatment PSA, and stage, overexpression of p16 did not contribute prognostic information [6].

In contrast, in another study including 104 patients, high level of p16 protein expression, quantitated by immunofluorescence flow cytometry, was an independent predictor of BF, although no significant association was found between p16 and standard clinico-pathological variables including serum pre-treatment PSA [19]. This finding was not sufficiently explicable even by the authors themselves as they observed no statistically significant association between p16 expression and BF in the same cohort when classic immunohistochemistry was used as a method of assessment [7].

A larger study of 206 patients with clinically localized PC evaluated p16 immunohistochemistry in areas of high-grade intraepithelial neoplasia (HGPIN) and of cancer in the same specimen. In the cancers, p16 overexpression, defined as either > 1% or > 5% nuclear staining, was associated with stage and disease relapse but did not correlate with age, pre-treatment PSA concentration, or Gleason score. In a multivariate model, overexpression of p16INK4A in HGPIN was an independent predictor of disease relapse. Although 38 of the 206 patients in this group received neo-adjuvant hormone therapy (NHT) prior to surgery, this did not seem to influence the prognostic value of p16 expression when they were excluded from the analyses [20]. In our study we did not concurrently evaluate the p16 staining status of HGPIN in our tumor samples, thus our results are not directly comparable with these of Henshall *et al. *Even in this case, they do not mention whether p16 expression in areas of cancer alone independently correlated with relapse-free survival.

Numerous studies have examined the prognostic significance of p27 immunohistochemical expression in RPs, with both negative and positive results. In an early work, low p27 expression, classified as < 50% of cells p27 positive, correlated with a number of prognostic morphologic features (including Gleason score, positive surgical margins, seminal vesicle involvement, lymph node metastasis and tumor aneuploidy) but did not correlate with sub-clinical biochemical failure, concurring with our results [21]. Similarly, Erdamar *et al. *did not find any association between the mean labeling index (LI) of p27Kip1 expression in cancers (LI: 43.5 +/-3.7%, defined as the percentage of p27-positive cells among epithelia of the same category) and Gleason score, stage or disease progression after RP [22]. In another study, decreased p27Kip1 staining (defined as < 25% of nuclei stained positive for p27Kip1) correlated with higher Gleason grade and was an independent predictor of treatment failure in the node-negative cohort. However, p27 was not an independent prognostic factor when 24 of 113 patients who underwent pre-operative NHT were excluded from the analysis [23]. Yang *et al. *found that absence of p27kip1 expression was the strongest predictor of biochemical relapse in patients with clinically organ confined disease [24]. In another cohort, < 10% reactivity for p27Kip1 proved to be the only independent prognostic factor for the PSA recurrence-free survival of 95 and 86 patients respectively [25,26]. These studies do not directly contradict our results, given the differences in cut-off values examined for positivity. A negative staining reaction as a predictor of recurrence-free survival did not achieve statistical significance at other cut-off values (</≥ 40 and </≥ 60% positivity) calculated, which were much closer to the one used in our study [25].

Cote *et al. *demonstrated the prognostic value of decreased p27Kip1 expression (cut-off value of 10%) for both the recurrence-free and overall survival of 96 PC patients undergoing RP. However, only stage C patients were evaluated [27].

At a cut-off level much lower than ours (30% positive cells), patients with low p27 expression showed a higher risk of biochemical relapse than the others, which maintained its predictive value in a multivariate analysis along with stage. However, only a small number of patients (47) with available follow up were examined [28].

Expression of p27 below a median value of 64% in tissue microarrays of 104 patients treated with RP was associated with high stage, elevated pre-operative PSA and time to biochemical failure. However, neither p27 alone nor combined PTEN/p27 expression retain their significance in multivariate analysis [29]. An artifact of loss of p27 expression during tissue processing in at least part of the samples of this study remains a possibility and might explain the different results supported by our and other groups using individual sample slides.

A negative association of p27 (Kip1) expression with tumor stage and grade has been reported in a group of 30 PC patients undergoing transurethral resection of the prostate (TURP) or RP. However, differences in sample size (only 30 PCs), surgical technique (80% perineal prostatectomy), immunohistochemical evaluation (whole tissue area), follow up (only 21 months) and cases of biochemical recurrence (only 7%) do not allow direct comparison between this study and ours [30]. Likewise, the reported high frequency of cytoplasmic p27 localization in high grade tumors compared to low-grade ones [31] cannot be confirmed by our results, as we performed only nuclear p27 IHC assessment, consistent with the majority of previous studies.

In concordance with our results, lack of association between expression of p27 or its ubiquitin ligase, Skp2, and time to PSA recurrence was reported in a prospective study of 162 African-American patients with clinically localized PC with a cut point for p27 set at < 40% [32]. The most recent study of p27 expression after RP in 100 cases of clinically localized PC, also failed to detect any association of the former with biochemical recurrence, although p27 positivity defined as ≥ 40% correlated well with a lower median pre-operative PSA and a lower Gleason score [33]. When p27 expression was examined in a cohort of 53 patients with pT2 stage disease, no correlation with Gleason score was revealed and p27 had no prognostic value in predicting biochemical relapse [34].

Another Greek team retrospectively evaluated the prognostic relevance of p27 in 94 patients undergoing RP. They observed a statistically significant univariate correlation of p27(Kip1) expression, at a level below 30%, with high pre-operative PSA values and an increased likelihood of BF after RP. However these data were not confirmed in multivariate analysis [35].

In the present analysis, we did not observe the strong correlation between decreased p16 or p27 expression and PSA recurrence-free survival reported by a part of existing relevant studies. Possible explanations include the selection of patients representative of a specific stage in one study. Also, the inclusion of patients who received NHT prior to their RP does not consist a homogenous population let alone that this variable is known to influence time to PSA relapse after surgery. It is not clear whether race or different ethnic groups of patients contributed to the difference in the correlation with treatment outcome results.

Most importantly, different methodologies, pathologic material, and classification schemas for the evaluation of positivity were used to define p27 expression levels in other studies. With regard to the cut-off level for positivity, our intention was to achieve a high sensitivity for detection of p16 and p27 expression aberrations, based on the underlying biology. This explains why any positive p16 expression was considered as p16 overexpression whereas a high cut-off positivity of 70% was used to establish an early detection of p27 loss in PC tissues.

Several limitations of the present work should be acknowledged. The small number of patients included in our study does not permit to draw safe conclusions as 70 patients is a small number of patients in which to identify an association with outcomes unless the marker examined is very robust. Lack of compartmental p16, p27 evaluation and absence of representation of lowest Gleason scores (2, 3, 4) in the study population might have also influenced the validity of our results. The group of patients studied in this report may be too homogeneous without sufficient events to be significant in the time period examined. Moreover, the prognostic values of these markers might have been better established if they had been compared to predicted outcomes of validated nomograms. Finally, our study was not intended to be all-inclusive of current prognostic markers such as surgical margins, seminal vesicle invasion, tumor marker ploidy status and proliferation indices.

## Conclusions

Our study was designed in an effort to evaluate the utility of p16 and p27 assessment in the clinical course of PC patients. The presence or absence of their significant correlations with established clinical variables as well as of their interrelations need to be examined in a large prospective cohort, taking into consideration all previous discordances.

## Competing interests

The authors declare that they have no competing interests.

## Authors' contributions

GM and CNP designed the study. GK and GM performed the radical prostatectomies and collected clinical data. FK and KK performed immunohistochemical evaluation. PJV collected clinical data and drafted the manuscript. GM, IG, DDD and CNP revised the manuscript. All authors read and approved the final manuscript.
